# Improved in Silico Identification of Protein‐Protein Interactions Using Deep Learning Approach

**DOI:** 10.1049/syb2.70008

**Published:** 2025-04-24

**Authors:** Irfan Khan, Muhammad Arif, Ali Ghulam, Somayah Albaradei, Maha A. Thafar, Apilak Worachartcheewan

**Affiliations:** ^1^ Department of Computer Science Abdul Wali Khan University Mardan KPK Mardan Pakistan; ^2^ College of Science and Engineering Hamad Bin Khalifa University Doha Qatar; ^3^ Information Technology Centre Sindh Agriculture University Tandojam Pakistan; ^4^ Department of Computer Science Faculty of Computing and Information Technology King Abdulaziz University Jeddah Saudi Arabia; ^5^ Department of Computer Science College of Computers and Information Technology Taif University Taif Saudi Arabia; ^6^ Department of Community Medical Technology Faculty of Medical Technology Mahidol University Bangkok Thailand

**Keywords:** medical information systems, proteomics, query processing, radial basis function networks

## Abstract

Protein–protein interactions (PPIs) perform significant functions in many biological activities likewise gene regulation, metabolic pathways and signal transduction. The deregulation of PPIs may cause deadly diseases, such as cancer, autoimmune, pernicious anaemia etc. Detecting PPIs can aid in elucidating the cellular process's underlying molecular mechanisms and contribute to facilitating the discovery of new proteins for the development of novel drugs. Although high‐throughput wet‐lab technologies have been matured to identify large scale PPI identification; however, the traditional experimental methods are costly and slow and resource intensive. To support experimental techniques, numerous computational approaches have been emerged for identifying PPIs solely from protein sequences. However, the performance of available PPI tools are unsatisfactory and gaps remain for further improvement. In this study, a novel deep learning‐based model, Deep_PPI, was developed for predicting multiple species PPIs. To extract the biological features, the authors used 21D vector representing 20 kinds' native and one special amino acid residue and implemented the Keras binary profile encoding technique to formulate each residue in proteins. The binary profile use the PaddVal strategy to equalise the length of positive and negative PPIs. After extracting the features, the authors fed them into one dimension convolutional neural network to build the final prediction model. The proposed Deep_PPI model, which consider the protein pairs into two convolutional heads. Finally, the authors concatenated the two outputs were concatenated from two branches concatenated by fully connected layer. The efficiency of the proposed predictor was demonstrated both on the cross validation and tested on various species datasets, for example, that is (Human, *C. elegans*, *E. coli*, and *H. sapiens*). The proposed model surpassed both the machine‐learning models and existing state‐of‐the‐art PPI methods. The proposed Deep_PPI will serve as valuable tool in the discovery of large‐scale PPIs in particular and provide insights for drugs development in general.

## Graphical abstract

Protein–protein interactions (PPIs) perform significant functions in many biological activities likewise gene regulation, metabolic pathways and signal transduction. The deregulation of PPIs may cause deadly diseases, such as cancer, autoimmune, pernicious anaemia etc. Detecting PPIs can aid in elucidating the cellular process's underlying molecular mechanisms and contribute to facilitating the discovery of new proteins for the development of novel drugs. Although high‐throughput wet‐lab technologies have been matured to identify large scale PPI identification; however, the traditional experimental methods are costly and slow and resource intensive. To support experimental techniques, numerous computational approaches have been emerged for identifying PPIs solely from protein sequences. However, the performance of available PPI tools are unsatisfactory and gaps remain for further improvement. In this study, a novel deep learning‐based model, Deep_PPI, was developed for predicting multiple species PPIs. To extract the biological features, the authors used 21D vector representing 20 kinds' native and one special amino acid residue and implemented the Keras binary profile encoding technique to formulate each residue in proteins. The binary profile use the PaddVal strategy to equalise the length of positive and negative PPIs. After extracting the features, the authors fed them into one dimension convolutional neural network to build the final prediction model. The proposed Deep_PPI model, which consider the protein pairs into two convolutional heads. Finally, the authors concatenated the two outputs from two branches concatenated by fully connected layer.

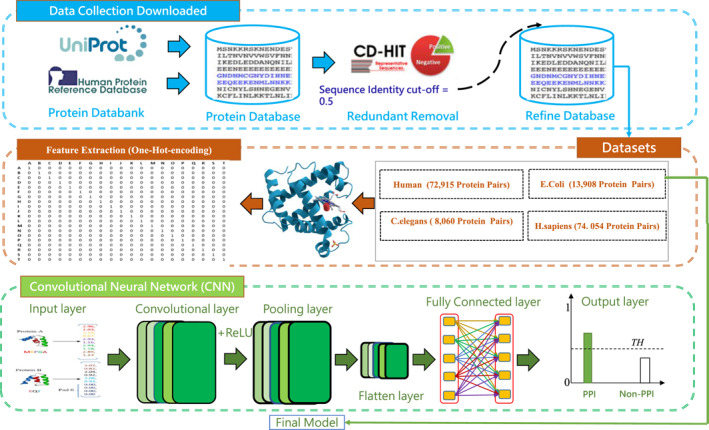

## 1 Introduction

In proteomics, proteins are complicated macromolecule participate virtually in every cellular process, such as material transportation, signal transduction, and metabolic cycles [1]. Usually, they exert its particular molecular function through interaction with other components instead of working alone [2, 3, 4]. Therefore, study about protein‐protein interactions (PPIs) or interactome have caught increasing interest from researcher in the investigation of various species [5]. PPIs exist ubiquitously and perform a vital role in a wide range of biological activities at molecular level likewise information transmission, protein localisation, DNA replication, post‐translation modification, apoptosis, cell growth and proliferation [6, 7, 8]. The past research demonstrate that the abnormality of PPI seriously disturb their appropriate function and may lead many dangerous diseases including cancer, autoimmune disease, cardiovascular disease, neurodegenerative disorders, pernicious anaemia [9]. Furthermore, because of its significance in life science, PPI is closely related to explore the mechanisms of human disease, prevention, and treatment. In this regard, the accurate prediction of PPIs is a critical step in the discovery of new drug targets and drug designing. Although, considerable technological improvement have been attempted to annotate the large scale uncovered interaction between proteins. These myriad wet‐laboratory (in vivo or in vitro) methods for example, mass spectrometry [10], mass spectrometry‐based (MS) screening and analysis of PPI [11], yeast to hybrid (Y2H) screen [12], tandem affinity purification (TAP) [13], protein chip technology [14], and other reliable biological methods [15] are successfully applied to investigate PPIs. However, the high experimental cost, slow process and intensive resource restrict them inapplicable due accumulation of protein samples accumulated in the post‐genomic era [16]. Therefore, the silico‐based approaches are desperately needed to complement the limitation of traditional biochemical techniques for accurate prediction of interacting protein pairs.

Over the last decades, we have witnessed remarkable contribution of computational‐based prediction of PPI solely from sequence information [17, 18, 19, 20, 21, 22, 23]. However, majority of the existing methods only considered traditional machine learning‐based models and the overall prediction performance is limited.

**FIGURE 1:**
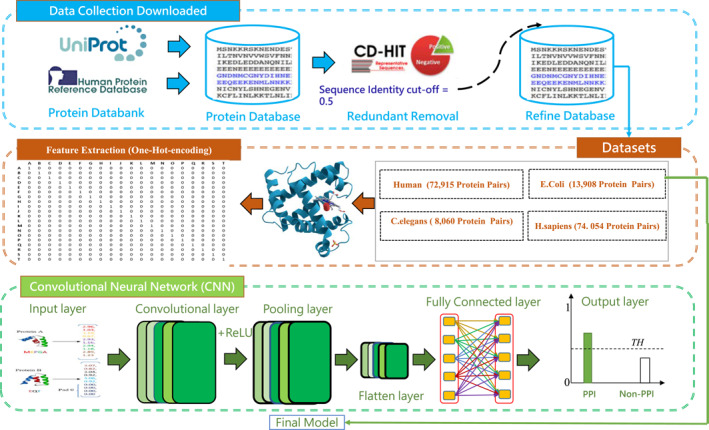
Framework of the proposed Deep_PPI model.

The contribution of this work is threefold: feature learning, model construction and performance evaluation. The schematic workflow and the proposed Deep_PPI development process is shown in Figure 1. Firstly, in order to simplify the feature representation procedure of PPIs, we used the following two strategies. (1) For the positive and negative samples of PPIs with unequal lengths, we applied the PaddVal strategy to make sure each PPI pair having the same length. After experiments, it is verified that: when set the PaddVal value to the length of 90% percent protein sequences, the experimental results performed better than other values (such as 75%, 80%, 85%, and 95%). (2) 20 kinds of native and one special amino acid are respectively represented by a number of 1–21, and then encoded by Keras. one‐hot function. In the prediction stage of Deep_PPI model, one‐dimensional convolutional neural network (1D‐CNN) was developed by considering the protein pairs into two convolutional heads. Finally, we concatenated the two outputs from two branches concatenated by fully connected layer. The proposed method outperformed conventional ML models both on fivefold cross‐validation and independent test results. The experimental outcomes further revealed that our developed protocol for PPI prediction is an effective method compare to other PPIs predictors. ‘To develop a really useful sequence‐based statistical predictor for a biological system as reported in a series of recent publications (see, e.g. [24, 25, 26, 27]), one should observe the Chou's 5‐step rule [28]; that is, making the following five steps very clear: (i) benchmark dataset; (ii) biological sequence formulation; (iii) operation engine/algorithm; (iv) cross‐validation; (v) web‐server establishment’.

## 2 Materials and Methods

In order to develop a robust computational method, it is essential to either construct or collect a high quality benchmark dataset that can be effectively used to train the prediction model [29, 30, 31]. The present research, considered the following publicly available datasets as follows:

### 2.1 Human and External PPIs Datasets

1. **Human PPIs dataset**: In this study, the human dataset was provided by Pan et al. [17], and extracted from YuLab (http://www.csbio.sjtu.edu.cn/bioinf/LR_PPI/Data.htm). There are 36,630 positive (taken from Human Protein Reference Database v2007) and 36,480 negative protein pairs (from Swiss‐Prot v57.3) in the human PPI dataset. As described in Pan et al. [17], after deleting protein pairs with abnormal amino acids (B, J, O, U, X, Z) and also those pairs less than 50AA, eventually, the human PPI dataset comprised of 36,591 positive and 36,324 negative protein interaction pairs.
2. **Other three PPIs datasets**: Excepting the aforementioned Human PPI dataset, we also adopted another three datasets, including *Caenorhabditis elegans* (i.e. *C. elegans*), *Escherichia coli* (i.e., *E. coli*), and *Homo sapiens* (i.e. *H. sapiens*), (stored in http://cic.scu.edu.cn/bioinformatics/predict_PPI/default.html), to verify the effectiveness of the proposed Deep_PPI model. As listed in Table 1, the *C. elegans* dataset consists of 8060 protein interaction pairs (including 4030 negative and 4030 positive protein pairs), the *E. coli* comprise of 6954 positive pairs and 6954 negative pairs (totally 13,908 protein pairs), and the *H. sapiens* contains a total of 74,054 protein pairs (37,027 positive pairs and 37,027 negative pairs).

### 2.2 Feature Learning Using Tokenisation and One‐Hot Encoding

Feature representation is an important phase to encode the biological PPI protein sequences into numerical feature vectors [32, 33, 34].

For the aforementioned four benchmark datasets, we utilised the tokenisation and ‘PaddVal’ technology to ensure the same length of positive and negative PPI samples. Tokenisation is an important technique for enhancing the interpretability of the data as input to any deep learning model [35]. Then, we take the length of 90% percent proteins as the value of PaddVal, add zero if one protein's length is less than this value and cut one protein if its length larger than the PaddVal. After several experiments, we set the PaddVal values to 887, 715, 1154, and 1141 for the *C. elegans*, *E. coli*, *H. sapiens*, and Human datasets, respectively.

For any residues in one protein, we adopted the keras. One‐hot function to encode each residue in one protein. After such feature representation, we transformed the information of amino acid residues in protein into numerical information. After converting the tokenised padded sequences into numerical feature vector the proposed Deep_PPI model, were trained on the following parameters as given in Table 2.

**TABLE 1:** Statistical information of benchmark datasets.

Dataset name	Number of positive protein pairs	Number of negative protein pairs	Total PPI pairs
Human	36,630	36,480	72,915
*C. elegans*	4030	4030	8060
*E. coli*	6954	6954	13,908
*H. sapiens*	37,027	37,027	74,054

**TABLE 2:** Recommended parameter of DEEP_PPI model in experiments.

Name	Parameter range	Recommendation
numFilters	[64, 128, 256, 512, 1024]	[128, 256, 512]
kernelSize	[2, 3, 5, 7]	2
actFun	[‘Relu’, ‘swish’]	‘Relu’
dropRate	[0.1, 0.2, 0.3]	0.1
fcNeurons	[128, 256, 512, 1024]	[128, 256, 512]
numConvLayers	[1, 2, 3, 4, 5]	3
numFcLayers	[1, 2, 3, 4, 5]	3
learnRate	[0.001, 0.01]	0.001

### 2.3 Evaluation Indices

Several evaluation indicators were utilised to illustrate effectiveness of models [32, 36, 37, 38], including accuracy (ACC), sensitivity (Sen) or Recall, specificity (Spe), F1‐score (F1), error rate (ER), precision (Pre), false‐positive ‐rate (FPR), Matthews‐correlation‐coefficient (MCC), false‐negative‐rate (FNR), and negative‐predictive‐value (NPV) defined below:

(1) Spe=TN/(TN+FP),0≤Spe≤1

(2) Pre=TP/(TP+FP),0≤Pre≤1

(3) FPR=FP/(TN+FP),0≤FPR≤1

(4) FNR=FN/(TP+FN),0≤FNR≤1

(5) NPV=TN/(TN+FN),0≤NPV≤1

(6) ER=FP/(TP+TN+FP+FN),0≤ER≤1

(7) $$F_{1}=2\times TP\;/\left(2\times TP+FP+FN\right),0\leq F_{1}\leq 1$$

(8) Recall/Sen=TP/(TP+FN),0≤Recall/Sen≤1

(9) ACC=(TP+TN)/(TP+TN+FP+FN),0≤ACC≤1

(10) $$MCC=\left(TP\times TN-FP\times FN\right)/\sqrt{\left(TP+FP\right)\left(TP+FN\right)\left(TN+FP\right)\left(TN+FN\right)},-1\leq MCC\leq 1$$

where in the above equations ((1), (2), (3), (4), (5), (6), (7), (8), (9), (10)), *TP* (true‐positive) denotes the true predictions of PPI pairs and *TN* (true negative) denotes the true prediction of non‐PPI pairs; *FN* (false‐negative) represent the wrong negative prediction; *FP* (false‐positive) represent the wrong prediction of positive sequences.

## 3 The Design and Parameter Optimisation of Deep_PPI Model

### 3.1 The Architecture of Deep_PPI Model

**FIGURE 2:**
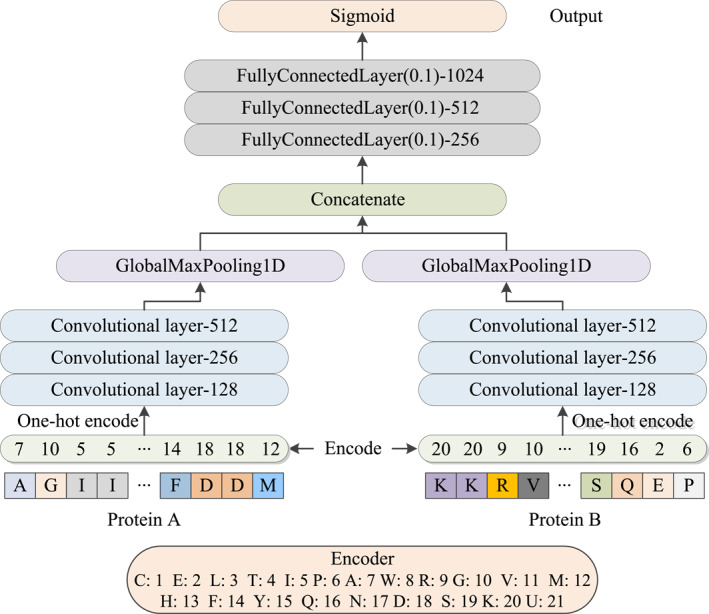
Architecture of the proposed Deep_PPI.

Deep learning (DL) approaches have been emerged in wide range bioinformatics and computational problems for analysing and predicting protein's functions such as prediction of ubiquitin–proteasome pathway (UPP) [39], lysine crotonylation sites [40], and non‐synonymous single‐nucleotide polymorphisms (nsSNPs) [27, 30]. Inspired by this, we proposed a DL model for identifying and characterising various types of PPIs. The workflow of the newly proposed Deep_PPI model (deep learning model for protein‐protein interactions) is depicted in Figure 2. It comprised of three mainly parts. Firstly, for one pair positive and negative protein interaction pair (namely Protein A and Protein B), we utilised 1–21 to represent each residue in proteins and then encoded the numerical information into 0–1 form using Keras. one‐hot function. In the second part, ‘Protein A’ and ‘Protein B’ passed through three groups of convolutional layers (with neuron = 128, 256, and 512) and one GlobalMaxPooling1D layer. Then, concatenate the outputs of the aforementioned two paths and followed by three fully connected layers (with dropout = 0.1 and neuron = 256, 512, and 1024). Thirdly, we utilised the sigmoid function to provide the prediction output.

During Deep_PPI model training procedure, we used Grid Search method to select the optimal parameters and listed the search range of parameters in Table 2. Specifically, we set the number of filters (namely numFilters) in [64, 128, 256, 512, 1024], kernel size (named as kernelSize) in [2, 3, 5, 7], activate function (called actFun) in [‘relu’,‘swish’], dropout rate (termed as dropRate) in [0.1, 0.2, 0.3], fully connected neurons (namely fcNeurons) in [128, 256, 512, 1024], number of convolutional layer (named as numConvLayers) in refs. [1, 2, 3, 4, 5], number of fully connected layers (called numFcLayers) in refs. [1, 2, 3, 4, 5], and learning rate in [0.001, 0.01]. After grid‐search verifying, we set the relatively optimal parameters (shown in the ‘Recommendation’ column of Table 2) for Deep_PPI model on four PPIs datasets.

In addition, to overcome the over‐fitting of the proposed DL‐based model, we set early stopping strategy during the Deep_PPI model training procedure. Specifically, we monitor the value of ‘val_acc’ during model training. If the ‘val_acc’ value does not increase after three cycles, the training procedure is terminated and the corresponding parameters with the maximum value of ‘val_acc’ are saved, which are used to test the model.

## 4 Results and Discussions

### 4.1 The Prediction Performance of Deep_PPI Model on Four Datasets

**FIGURE 3:**
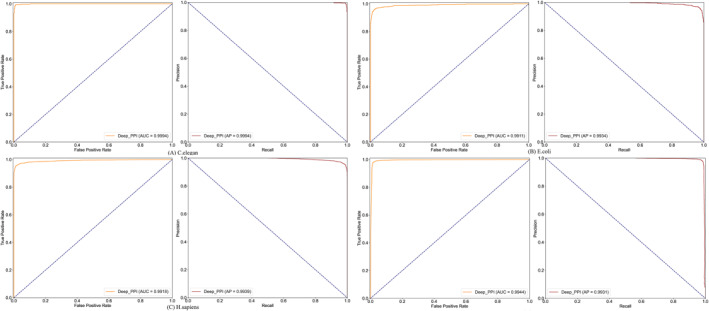
ROC and recall‐precision curve of Deep_PPI on four datasets.

In the present sub‐section, we demonstrated the prediction efficacy of the proposed Deep_PPI model on four benchmark PPIs datasets. Before doing experiments, we split each dataset into a training data part (by 90%) and independent test data part (by 10%). On the training data, we adopted the Grid Search trip (parameters range setting listed in Table 2) to find the optimal parameters group and trained the Deep_PPI model. After parameters searching (recommended parameters documented in the ‘Recommendation’ column of Table 2) and training procedure, we tested Deep_PPI model on the unseen test samples of each benchmark dataset. Based on the prediction outcomes, we calculated the corresponding evaluation indices (defined in Section 2.3), which were listed in Tables 3 and 4, and also displayed in Figure 3A–D.

**TABLE 3:** Prediction performance of the Deep_PPI model on the test data of four datasets.

Dataset	*MCC*	*ACC*	*Recall/Sen*	*Spe*	*Pre*	*NPV*	*F*_*1*_	*AUC*
*C. elegans*	0.9801	0.9901	0.9929	0.9869	0.9883	0.9921	0.9906	0.9994
*E. coli*	0.9341	0.9669	0.9565	0.9779	0.9784	0.9554	0.9673	0.9911
*H. sapiens*	0.9412	0.9703	0.9524	0.9881	0.9877	0.9541	0.9697	0.9918
Human	0.9688	0.9844	0.9883	0.9805	0.9808	0.9881	0.9845	0.9944

**TABLE 4:** Confusion matrix and three types of errors of the Deep_PPI model on the test data of four datasets.

Dataset	*TP*	*TN*	*FP*	*FN*	*ER*	*FPR*	*FNR*
*C. elegans*	421 (52.23%)	377 (46.77%)	5 (0.62%)	3 (0.37%)	0.0062	0.0131	0.0071
*E. coli*	681 (48.96%)	664 (47.74%)	15 (1.08%)	31 (2.23%)	0.0108	0.0221	0.0435
*H. sapiens*	3525 (47.60%)	3661 (49.43%)	44 (0.59%)	176 (2.38%)	0.0059	0.0119	0.0476
Human	3632 (49.68%)	3565 (48.76%)	71 (0.97%)	43 (0.59%)	0.0097	0.0195	0.0117

By carefully observing the all performance measures such *TN,FP,TP,* and *FP* predictions in Table 4, it is easily found that the newly proposed Deep_PPI model are quite effective for PPIs prediction on four benchmark datasets. Take the *C. elegans* dataset for example, the Deep_PPI could achieved *TP* of 421 (by 52.23%) and *TN* of 377 (by 46.77%), only *FP* of 5 (0.62%) and *FN* of 3 (0.37%). Furthermore, Deep_PPI could get the *MCC*, *ACC*, and *AUC* values of 0.9801, 0.9901, and 0.9994. By regarding other evaluation indices (i.e. *ER*, *FPR*, *FNR*, *ACC*, *Recall*/*Sen*, *Spe*, *Pre*, *NPV*, and *F*_*1*_) and also on other three datasets (i.e. *E. coli*, *H. sapiens*, and Human), it can be also concluded that the newly proposed Deep_PPI is an effective model to predict PPIs.

### 4.2 Comparison Deep_PPI Model With Two Traditional Machine Learning Methods

**FIGURE 4:**
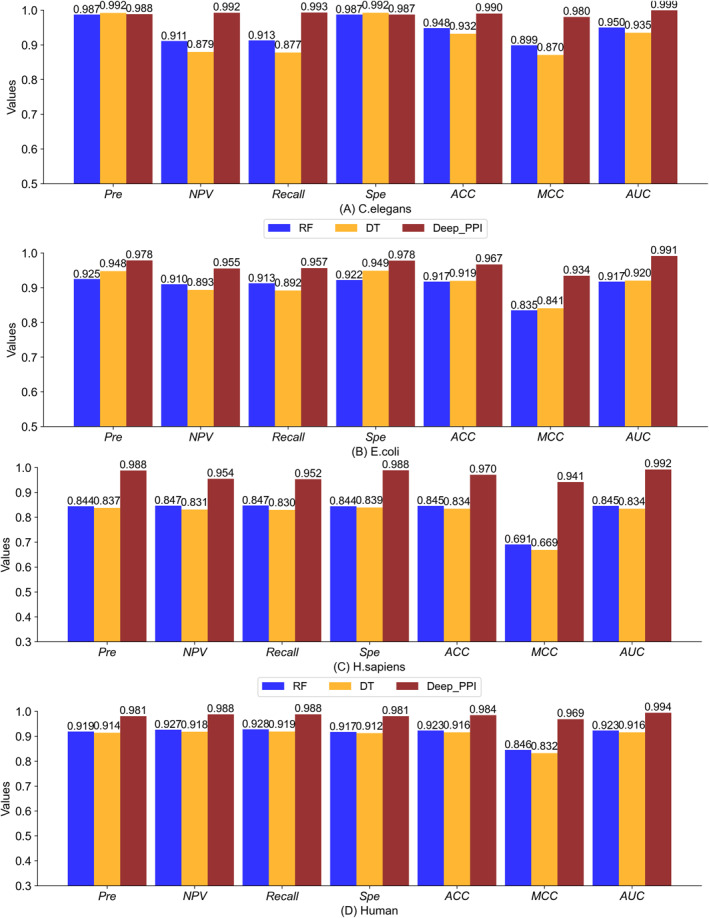
The *Pre*, *NPV*, *Recall*, *Spe*, *ACC*, *MCC*, and *AUC* values comparison of RF, DT, and Deep_PPI.

In the present subsection, we compared the traditional ML classifiers, random forest (RF) and decision tree (DT) with the proposed Deep_PPI model to describe the effectiveness of the developed method. The ML models were also evaluated, by feeding the same feature representation on independent (blind test) data of four benchmark datasets. The comparison results were depicted in Figure 4A–D and also documented in Table 5.

**TABLE 5:** The confusion matrix and three types of errors of RF, DT, and Deep_PPI on the test data of four datasets.

Dataset	Method	*TP*	*TN*	*FP*	*FN*	*ER*	*FPR*	*FNR*
*C. elegans*	RF	387 (48.01%)	377 (46.77%)	5 (0.62%)	37 (4.59%)	0.0062	0.0131	0.0873
	DT	372 (46.15%)	379 (47.02%)	3 (0.37%)	52 (6.45%)	0.0037	0.0079	0.1226
	Deep_PPI	421 (52.23%)	377 (46.77%)	5 (0.62%)	3 (0.37%)	0.0062	0.0131	0.0071
*E. coli*	RF	650 (46.73%)	626 (45.00%)	53 (3.81%)	62 (4.46%)	0.0381	0.0781	0.0871
	DT	635 (45.65%)	644 (46.30%)	35 (2.52%)	77 (5.54%)	0.0252	0.0515	0.1081
	Deep_PPI	681 (48.96%)	664 (47.74%)	15 (1.08%)	31 (2.23%)	0.0108	0.0221	0.0435
*H. sapiens*	RF	3134 (42.23%)	3127 (42.22%)	578 (7.80%)	567 (7.66%)	0.0780	0.1560	0.1532
	DT	3070 (41.45%)	3109 (41.98%)	596 (8.05%)	631 (8.52%)	0.0805	0.1609	0.1705
	Deep_PPI	3525 (47.60%)	3661 (49.43%)	44 (0.59%)	176 (2.38%)	0.0059	0.0119	0.0476
Human	RF	3411 (46.66%)	3335 (45.62%)	301 (4.12%)	264 (3.61%)	0.0412	0.0828	0.0718
	DT	3378 (46.20%)	3318 (45.38%)	318 (4.35%)	297 (4.06%)	0.0435	0.0875	0.0808
	Deep_PPI	3632 (49.68%)	3565 (48.76%)	71 (0.97%)	43 (0.59%)	0.0097	0.0195	0.0117

By analysis the success rates depicted in Figure 4, we can observe that Deep_PPI model is superior to RF and DT on all four datasets. More specifically, on the *C. elegans* (Figure 4A), *E. coli* (Figure 4B), *H. sapiens* (Figure 4C), and Human dataset (Figure 4D), the predicted values of *MCC* and *AUC* of Deep_PPI are 0.980 and 0.999, 0.934 and 0.991, 0.941 and 0.992, 0.969 and 0.994. Whereas, the corresponding *MCC* and *AUC* values of RF are 0.899 and 0.950, 0.835 and 0.917, 0.691 and 0.845, 0.846 and 0.923. Whereas, the corresponding *MCC* and *AUC* values of RF are 0.870 and 0.935, 0.841 and 0.920, 0.669 and 0.834, 0.832 and 0.916. Obviously, Deep_PPI can obtain much higher prediction values of *MCC* and *AUC* than both classifiers DT and RF.

Such superiority of the Deep_PPI model are also appeared as the *TP*, *TN*, *FP*, *FN*, *ER*, *FPR*, and *FNR* values in Table 5. As listed in Table 5, Deep_PPI can target more accurate positive and negative PPIs with less error rate, and also smaller *ER*, *FPR*, *FNR*, than RF and DT. For instance, on the *C. elegans* dataset, Deep_PPI can obtain *TP* of 421 (52.23%), *TN* of 377 (46.77%), *FP* of 5 (0.62%), *FN* of 3 (0.37%), *ER* of 0.0062, *FPR* of 0.0131, and *FNR* of 0.0071, while RF get *TP* of 387 (48.01%), *TN* of 377 (46.77%), *FP* of 5 (0.62%), *FN* of 37 (4.59%), *ER* of 0.0062, *FPR* of 0.0131, and *FNR* of 0.0873, whereas DT can predict *TP* of 372 (46.15%), *TN* of 379 (47.02%), *FP* of 3 (0.37%), *FN* of 52 (6.45%), *ER* of 0.0037, *FPR* of 0.0079, and *FNR* of 0.1226. In summary, the newly proposed Deep_PPI model is a better choice in PPIs prediction than two traditional machine learning methods RF and DT.

### 4.3 Verifying Deep_PPI Model Evaluation via CV and Independent Testing Method

#### 4.3.1 Implement the Independent Test Repeating Five Times

In the deep learning technology‐based Deep_PPI model, there are some random initialisation parameters in the model training procedure. In order to verify the stability and robustness of Deep_PPI, we trained it five times on the training data (by 90%), and correspondingly, tested it on the independent test data (by 10%) five times. Meanwhile, we recorded the corresponding evaluation indices of prediction results in Table 6 and Table S1 of the supplementary material file (namely ‘Times = 1’, ‘Times = 2’, ‘Times = 3’, ‘Times = 4’, and ‘Times = 5’ row). The ‘Overall’ row in Table 6 and Table S1 is the mean and standard deviation of the corresponding evaluation indicators.

**TABLE 6:** Prediction performance of Deep_PPI on the independent test data of four datasets (five times).

Dataset	Times	*MCC*	*ACC*	*Recall/Sen*	*Spe*	*Pre*	*NPV*	*F*_*1*_	*AUC*
*C. elegans*	Times = 1	0.9801	0.9901	0.9929	0.9869	0.9883	0.9921	0.9906	0.9994
	Times = 2	0.9801	0.9901	0.9882	0.9921	0.9929	0.9870	0.9905	0.9997
	Times = 3	0.9605	0.9801	0.9693	0.9921	0.9928	0.9668	0.9809	0.9994
	Times = 4	0.9778	0.9888	0.9811	0.9974	0.9976	0.9794	0.9893	0.9993
	Times = 5	0.9776	0.9888	0.9906	0.9869	0.9882	0.9895	0.9894	0.9997
	Overall	0.9752 ± 0.0083	0.9876 ± 0.0042	0.9844 ± 0.0095	0.9911 ± 0.0044	0.9919 ± 0.0039	0.9830 ± 0.0102	0.9881 ± 0.0041	0.9995 ± 0.0002
*E. coli*	Times = 1	0.9311	0.9655	0.9593	0.9720	0.9729	0.9579	0.9661	0.9912
	Times = 2	0.9329	0.9662	0.9522	0.9809	0.9812	0.9514	0.9665	0.9913
	Times = 3	0.9338	0.9669	0.9677	0.9661	0.9677	0.9661	0.9677	0.9920
	Times = 4	0.9282	0.9641	0.9579	0.9705	0.9715	0.9565	0.9646	0.9923
	Times = 5	0.9341	0.9669	0.9565	0.9779	0.9784	0.9554	0.9673	0.9911
	Overall	0.932 ± 0.0024	0.9659 ± 0.0012	0.9587 ± 0.0057	0.9735 ± 0.0059	0.9744 ± 0.0054	0.9575 ± 0.0054	0.9664 ± 0.0012	0.9916 ± 0.0005
*H. sapiens*	Times = 1	0.9347	0.9672	0.9543	0.9800	0.9795	0.9555	0.9667	0.9931
	Times = 2	0.9381	0.9688	0.9533	0.9843	0.9838	0.9547	0.9683	0.9923
	Times = 3	0.9295	0.9646	0.9535	0.9757	0.9751	0.9546	0.9642	0.9909
	Times = 4	0.9416	0.9706	0.9541	0.9870	0.9866	0.9556	0.9701	0.9921
	Times = 5	0.9320	0.9657	0.9476	0.9838	0.9832	0.9495	0.9651	0.9921
	Overall	0.9352 ± 0.0048	0.9674 ± 0.0024	0.9526 ± 0.0028	0.9822 ± 0.0044	0.9816 ± 0.0044	0.9540 ± 0.0026	0.9669 ± 0.0024	0.9921 ± 0.0008
Human	Times = 1	0.9688	0.9844	0.9883	0.9805	0.9808	0.9881	0.9845	0.9965
	Times = 2	0.9614	0.9807	0.9834	0.9780	0.9783	0.9831	0.9809	0.9951
	Times = 3	0.9644	0.9822	0.9850	0.9794	0.9797	0.9848	0.9824	0.9959
	Times = 4	0.9620	0.9810	0.9815	0.9805	0.9807	0.9813	0.9811	0.9944
	Times = 5	0.9615	0.9807	0.9853	0.9761	0.9765	0.9850	0.9809	0.9946
	Overall	0.9603 ± 0.0060	0.9801 ± 0.0030	0.9824 ± 0.0044	0.9779 ± 0.0035	0.9781 ± 0.0034	0.9823 ± 0.0044	0.9802 ± 0.0030	0.9944 ± 0.0014

From the comparison results in Table 6, it can be seen that there is no much difference of Deep_PPI performance among ‘Times = 1’ to ‘Times = 5’. For example, on the *C. elegans* dataset, the ‘Overall’ values of *MCC*, *ACC* and *AUC* of Deep_PPI are 0.9752 ± 0.0083, 0.9876 ± 0.0042, and 0.9995 ± 0.0002. Regarding to other evaluation indices (i.e., *Recall*/*Sen*, *Spe*, *Pre*, *NPV*, and *F*_*1*_) and on other three datasets (i.e., *E. coli*, *H. sapiens*, and Human), the same phenomenon can also be seen. Except for the *MCC*, *ACC*, *Recall*/*Sen*, *Spe*, *Pre*, *NPV*, *F*_*1*_, *AUC* values of Deep_PPI on four datasets, we also listed the *TP*, *TN*, *FP*, *FN*, *ER*, *FPR*, and *FNR* values in Table S1. For more detailed information, please refer to Table S1.

#### 4.3.2 Verify via Five‐Fold Cross Validation

In this section, we verified the proposed Deep_PPI model via five‐fold cross validation. During such validation, each dataset is firstly divided into five equal parts. Then, train the Deep_PPI model on 4/5 parts of the data each time and test it on the left 1/5 part of the data. After five cycles, each part of data was used as the training set for four times and as the test set once. We documented the evaluation indices of prediction results each time (i.e. ‘Fold = 1’, ‘Fold = 2’, ‘Fold = 3’, ‘Fold = 4’, and ‘Fold = 5’ raw) and also calculated their average values (i.e. the ‘Overall’ raw) on the test data as documented in Table 7 and Table S2.

**TABLE 7:** Prediction performance of Deep_PPI via 5‐fold cross validation on four datasets.

Dataset	Fold	*MCC*	*ACC*	*Recall/Sen*	*Spe*	*Pre*	*NPV*	*F*_*1*_	*AUC*
*C. elegans*	Fold = 1	0.9634	0.9814	0.9628	1.0000	1.0000	0.9641	0.9810	0.9965
	Fold = 2	0.9487	0.9739	0.9529	0.9950	0.9948	0.9548	0.9734	0.9830
	Fold = 3	0.9628	0.9814	0.9801	0.9826	0.9826	0.9802	0.9814	0.9965
	Fold = 4	0.9585	0.9789	0.9603	0.9975	0.9974	0.9617	0.9785	0.9970
	Fold = 5	0.9555	0.9777	0.9677	0.9876	0.9873	0.9684	0.9774	0.9885
	Overall	0.9578 ± 0.0060	0.9787 ± 0.0031	0.9648 ± 0.0101	0.9926 ± 0.0072	0.9924 ± 0.0073	0.9658 ± 0.0094	0.9783 ± 0.0032	0.9923 ± 0.0063
*E. coli*	Fold = 1	0.9643	0.9820	0.9713	0.9928	0.9928	0.9718	0.9818	0.9940
	Fold = 2	0.9619	0.9806	0.9612	1.0000	1.0000	0.9626	0.9802	0.9992
	Fold = 3	0.9576	0.9784	0.9583	0.9986	0.9985	0.9599	0.9780	0.9883
	Fold = 4	0.9543	0.9770	0.9640	0.9899	0.9897	0.9650	0.9767	0.9921
	Fold = 5	0.9375	0.9684	0.9482	0.9885	0.9880	0.9503	0.9677	0.9901
	Overall	0.9551 ± 0.0106	0.9773 ± 0.0053	0.9606 ± 0.0084	0.994 ± 0.0051	0.9938 ± 0.0053	0.9619 ± 0.0079	0.9769 ± 0.0055	0.9927 ± 0.0042
*H. sapiens*	Fold = 1	0.9034	0.9514	0.9336	0.9692	0.9681	0.9359	0.9505	0.9809
	Fold = 2	0.9223	0.9607	0.9390	0.9824	0.9816	0.9415	0.9598	0.9898
	Fold = 3	0.8736	0.9367	0.9482	0.9252	0.9269	0.9469	0.9374	0.9821
	Fold = 4	0.9188	0.9590	0.9368	0.9811	0.9802	0.9395	0.9580	0.9876
	Fold = 5	0.8979	0.9487	0.9314	0.9660	0.9648	0.9337	0.9478	0.9842
	Overall	0.9031 ± 0.0225	0.9513 ± 0.0111	0.9388 ± 0.0070	0.9637 ± 0.0267	0.9634 ± 0.0255	0.9404 ± 0.0055	0.9508 ± 0.0104	0.9859 ± 0.0034
Human	Fold = 1	0.9680	0.9840	0.9825	0.9855	0.9855	0.9825	0.9840	0.9957
	Fold = 2	0.9579	0.9789	0.9787	0.9792	0.9792	0.9786	0.9790	0.9940
	Fold = 3	0.9683	0.9841	0.9861	0.9822	0.9823	0.9860	0.9842	0.9961
	Fold = 4	0.9540	0.9770	0.9784	0.9756	0.9758	0.9783	0.9771	0.9927
	Fold = 5	0.9609	0.9804	0.9863	0.9745	0.9749	0.9861	0.9806	0.9946
	Overall	0.9603 ± 0.0060	0.9801 ± 0.0030	0.9824 ± 0.0044	0.9779 ± 0.0035	0.9781 ± 0.0034	0.9823 ± 0.0044	0.9802 ± 0.0030	0.9944 ± 0.0014

As listed in Table 7, it is easily found that there is no much difference among ‘Fold = 1’ to ‘Fold = 5’. Take *C. elegans* dataset for example, the ‘Overall’ values of *MCC*, *ACC*, and *AUC* of Deep_PPI are 0.9578 ± 0.006, 0.9787 ± 0.0031, and 0.9923 ± 0.0063. In terms to other evaluation indices (i.e. *Recall*/*Sen*, *Spe*, *Pre*, *NPV*, and *F*_*1*_) and on other three datasets (i.e. *E. coli*, *H. sapiens*, and Human), we can also see similar phenomenon comparison results. Except for the *MCC*, *ACC*, *Recall*/*Sen*, *Spe*, *Pre*, *NPV*, *F*_*1*_, *AUC* values of Deep_PPI on four datasets, we also listed the *TP*, *TN*, *FP*, *FN*, *ER*, *FPR*, and *FNR* values in Table S2. For more detailed information, please refer to Table S2.

#### 4.3.3 Comparison of Training and Independent Test Results of Independent Test

**FIGURE 5:**
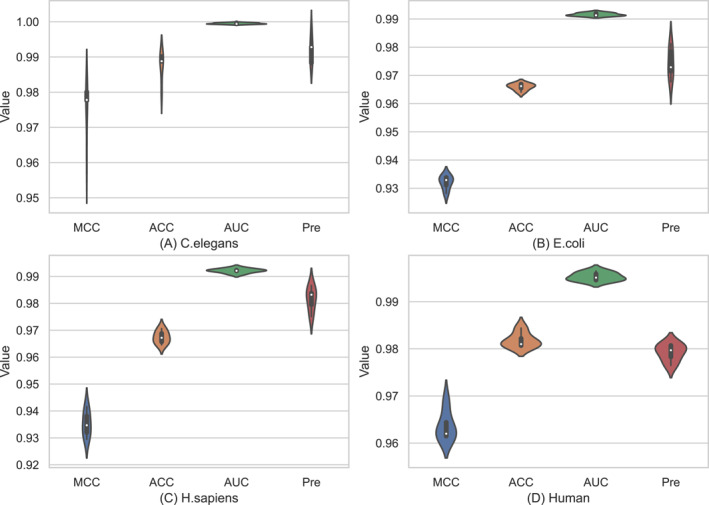
Violin plots of Deep_PPI via the independent test (from ‘Times = 1’ to ‘Times = 5’) in terms of four performance metrics.

**FIGURE 6:**
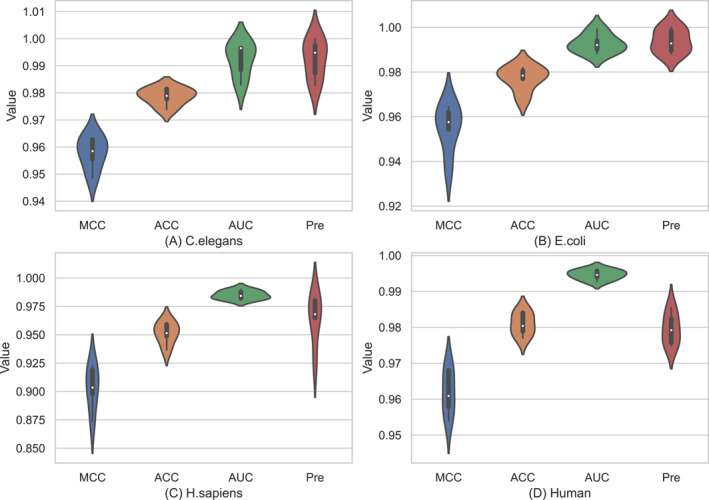
Violin plots of Deep_PPI via five‐fold cross validation (from ‘Fold = 1’ to ‘Fold = 5’) in terms of four performance metrics.

In order to more clearly observe the comparison between the empirical prediction outcomes in subsections 4.3.1 and Section 4.3.2, we drew the violin diagrams of four evaluation metrics (namely *MCC*, *ACC*, *AUC*, and *Pre*), illustrated in Figure 5 *C. elegans* (Figure 5A), *E. coli* (Figure 5B), *H. sapiens* (Figure 5C), and Human dataset (Figure 5D) and Figure 6 *C. elegans* (Figure 6A), *E. coli* (Figure 6B), *H. sapiens* (Figure 6C), and Human dataset (Figure 6D) depicted the results of the independent test (i.e. ‘Times = 1’ to ‘Times = 5’) and Figure 6A–D 5‐fold CV results of the training data (i.e. ‘Fold = 1’ to ‘Fold = 5’). Each violin chart could represent the maximum/minimum value, upper and lower quartile value, and median value of the corresponding evaluation indices.

Observing the subplots on each dataset in Figures 5 and 6, we can obtain the following conclusions:

1. It is easy to find that the evaluation indices depicted Figure 5 were generally greater than the corresponding value shown in Figure 6. For example, on the *C. elegans* dataset in Figure 5, the *MCC* was in range [0.9605, 0.9801] with the average value of 0.9752; the *ACC* was in [0.9801, 0.9901] with the average of 0.9876; the *AUC* is in [0.9993, 0.9997] with the average of 0.9995; and the *Pre* is in [0.9882, 0.9976] with the average of 0.9920. Correspondingly, as shown in Figure 6 (also on the *C. elegans* dataset), the *MCC*, *ACC*, *AUC*, and *Pre* values were in range [0.9487, 0.9634], [0.9739, 0.9814], [0.9830, 0.9970], and [0.9826, 1.0000], with the average values of 0.9578, 0.9787, 0.9923, and 0.9924, respectively.
2. By regarding to the *MCC* values, on the *H. sapiens* dataset, the proposed Deep_PPI model performed weakest via two evaluation modes among all four datasets. More specifically, when using the independent test mode, the *MCC* value of Deep_PPI is in range [0.9292, 0.9416] with the average value of 0.9352; When swift to the five‐fold cross‐validation mode, the *MCC* value of Deep_PPI is in range [0.8736, 0.9223] with an average value of 0.9032. The underlying reason for this phenomenon maybe that: the *H. sapiens* dataset comprised of 37,027 negatives and 37,027 positives, with a total of 74,054 protein‐protein interaction pairs, which is the largest dataset among the four benchmark datasets.
3. Under the independent test evaluation mode (results shown in Table 6 and Figure 5), the Deep_PPI model only tested the independent test data part of each dataset, accounting for 10%. Correspondingly, the success rates of 5‐fold CV (results documented in Table 7 and Figure 6) were the average of evaluation indices of Deep_PPI on the test data of each cycle. That is, the experimental results of five‐fold cross‐validation could evaluate each protein‐protein interaction pair in the dataset with no deviation. Relatively speaking, the five‐fold cross‐validation mode is a fairer way to compare Deep_PPI with existing methods. However, observing the results in Tables 6 and 7 and Figures 5 and 6, it is also clearly found that there is no much difference between the aforementioned two evaluation modes. Therefore, we used the results of Deep_PPI under two evaluation modes to compare with other methods in the following sections.

### 4.4 Comparison of the Deep_PPI Model With Existing Methods on Human Dataset

In the below subsection, we compared our proposed model with several existing methods such as SVM‐CTAC, Ada Boost (AB‐CTAC), Random Forest (RF‐CTAC) and DNN‐CTAC on the human dataset [17], as described in ‘Section 4.4.1’. ‘Section 4.4.2 compare of Deep_PPI with six existing methods on the Human dataset’.

#### 4.4.1 Comparison of Deep_PPI With Three Combinations of CTAC on the Human Dataset

In order to verify the validity of the proposed feature CTAC, the author replaced DNN with traditional machine learning classification techniques, such as SVM, Ada Boost (AB), and Random Forest (RF), accordingly formed SVM‐CTAC, AB‐CTAC, and RF‐CTAC. So, herein, we compared Deep_PPI with the aforementioned traditional machine‐learning classifiers, and the results were displayed in Table 8.

**TABLE 8:** Comparison results of Deep_PPI with SVM‐CTAC, AB‐CTAC, RF‐CTAC, and DNN‐CTAC on the Human dataset.

Classifiers	*Accuracy*	*Recall*	*AUC*
SVM‐CTAC	0.7453	0.7566	0.7451
AB‐CTAC	0.7238	0.7489	0.7237
RF‐CTAC	0.9793	0.9733	0.9795
DNN‐CTAC	0.9827	**0.9941**	0.9924
DeeP_PPI (the independent test‐5times^#^)	0.9818 ± 0.0016	0.9847 ± 0.0025	0.9953 ± 0.0009
DeeP_PPI (5‐fold cross validation^#^)	**0.9838 ± 0.0030**	0.9824 ± 0.0044	**0.9944 ± 0.0014**

*Note:* ‘the independent test‐5times^#^’ means the average values of the Deep_PPI model via the independent test five times (from ‘Times = 1’ to ‘Times = 5’), as the ‘Overall’ row of Table 6. ‘5‐fold cross validation^#^’ means the average values of ‘Fold = 1’ to ‘Fold = 5’ in the five‐fold cross validation. SVM‐CTAC, AB‐CTAC, and RF‐CTAC: by feeding the extracted CTAC feature vector into Support Vector Machine, Ada Boost, and Random Forest classifiers. The bold faced value denotes the high predictions in Table 8.

Observing the comparison results in Table 8, we found the following conclusions: (1) The prediction ability of SVM‐CTAC and AB‐CTAC were weaker than others, with the *accuracy*, *recall*, and *AUC* values of 0.7453, 0.7566, 0.7451, and 0.7238, 0.7489, 0.7237, respectively. (2) The prediction ability of RF‐CTAC was better than that of SVM‐CTAC and AB‐CTAC. Its *accuracy*, *recall*, and *AUC* values were 0.9793, 0.9733, and 0.9795, respectively, which were 0.2340, 0.2167, 0.2344 higher than the corresponding values of SVM‐CTAC, and 0.2555, 0.2244, 0.2558 higher than the corresponding values of AB‐CTAC. (3) DNN‐CTAC and Deep_PPI are methods built on deep learning technology, which could obtain much higher evaluation indices than RF‐CTAC. More specifically, the *AUC* values of DNN‐CTAC, Deep_PPI (the independent test‐5times), and Deep_PPI (5‐fold cross validation) were 0.9924, 0.9953 ± 0.0009, and 0.9944 ± 0.0014 respectively, which were 0.0129, 0.0158 ± 0.0009, and 0.0149 ± 0.0014 higher than the *AUC* value of RF‐CTAC. (4) By regarding to the *AUC* value, the Deep_PPI (the independent test‐5times) and Deep_PPI (5‐fold cross validation) were 0.0029 ± 0.0009 and 0.0020 ± 0.0014 higher than that of DNN‐CTAC, respectively.

As described in Section 3.1, the features used in Deep_PPI are simpler. More specifically, in the Deep_PPI model, the protein sequences processed by padding technology and encoded in 1–21 through the keras. one‐hot function, were fed into two paths (including positive and negative interaction sequences) for further feature processing. For larger‐scale protein‐protein interaction pairs data, this Deep_PPI model is easier to realise and takes less time to predict than DNN‐CTAC. Summarise the above results analyses, it could be concluded that Deep_PPI was superior to three traditional machine learning‐based methods (i.e. SVM‐CTAC, AB‐CTAC, and RF‐CTAC) and also performed better than deep learning technology‐based method (i.e. DNN‐CTAC).

#### 4.4.2 Comparison of Deep_PPI With Six Existing Methods on the Human Dataset

In this section, we compared Deep_PPI with seven existing PPIs prediction methods on the Human dataset. These methods include Shen's work (namely SVM‐CT) [18], Guo's work (termed as SVM‐AC) [19], Zhang's work (named SVM‐CS) [20], Huang's work (called DCT‐SMR) [21], Sun's work (including SAE‐AC and SAE‐CT) [22], and Wang's work (namely DNN‐CTAC) [23]. The average accuracy values of these methods were depicted in Figure 6.

**FIGURE 7:**
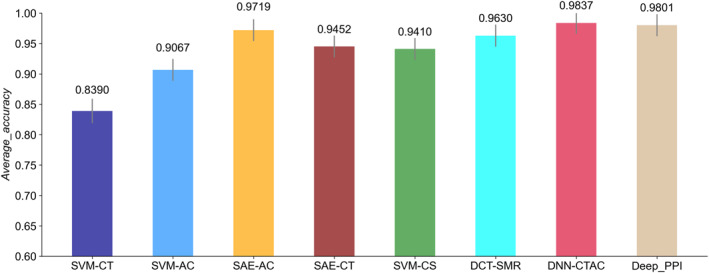
The average accuracy of Deep_PPI and other seven different methods on the Human dataset. The evaluation indices of seven existing PPIs prediction methods were taken from DNN‐CTAC [17, 23]. For more detailed information of abbreviation for method names, please refer to DNN‐CTAC [17, 23].

As shown in Figure 7, it is easily found that Deep_PPI outperforms SVM‐CT, SVM‐AC, ASE‐AC, SAE‐CT, SVM‐CS, and DCT‐SMR. Specifically, the average accuracy of SVM‐CT, SVM‐AC, ASE‐AC, SAE‐CT, SVM‐CS, and DCT‐SMR were 0.8390, 0.9067, 0.9719, 0.9452, 0.9410, and 0.9630, Deep_PPI could increase the average accuracy value to 0.9801, which were 0.1411, 0.0734, 0.0082, 0.0349, 0.0391, and 0.0171 higher than that of the aforementioned six methods. DNN‐CTAC and Deep_PPI could get the average accuracy values of 0.9837 and 0.9801, that is, DNN‐CTAC performed slightly higher than Deep_PPI on the Human dataset.

### 4.5 Comparison Deep_PPI With Existing Methods on *C. elegans*, *E. coli*, and *H. sapiens* Datasets

In this section, we compared Deep_PPI model with DNN_CTAC [17] on the *C. elegans*, *E. coli*, and *H. sapiens* datasets, comparison results listed in Table 9. Again, we listed the experimental results of Deep_PPI via two evaluation modes (including the independent test and five‐fold cross‐validation).

**TABLE 9:** Comparison performance of Deep_PPI with DNN_CTAC on *C. elegans*, *E. coli*, and *H. sapiens* datasets.

Method	Dataset	*Accuracy*	*Recall*	*AUC*
DNN_CTAC	*C. elegans*	0.9740 ± 0.0018	**0.9888 ± 0.0009**	0.9790 ± 0.0018
	*E. coli*	0.9641 ± 0.0012	**0.9809 ± 0.0028**	0.9726 ± 0.0033
	*H. sapiens*	0.9639 ± 0.0044	**0.9913 ± 0.0008**	0.9875 ± 0.0010
Deep_PPI	*C. elegans* (the independent test‐5times^#^)	**0.9876 ± 0.0042**	0.9844 ± 0.0095	**0.9995 ± 0.0002**
	*E. coli* (the independent test‐5times^#^)	**0.9659 ± 0.0012**	0.9587 ± 0.0057	**0.9916 ± 0.0005**
	*H. sapiens* (the independent test‐5times^#^)	**0.9674 ± 0.0024**	0.9526 ± 0.0028	**0.9921 ± 0.0008**
	*C. elegans* (5‐fold cross validation^#^)	**0.9787 ± 0.0031**	0.9648 ± 0.0101	**0.9923 ± 0.0063**
	*E. coli* (5‐fold cross validation^#^)	**0.9773 ± 0.0053**	0.9606 ± 0.0084	**0.9927 ± 0.0042**
	*H. sapiens* (5‐fold cross validation^#^)	**0.9513 ± 0.0111**	0.9388 ± 0.0070	**0.9859 ± 0.0034**

*Note:* ‘the independent test‐5times^#^’ means the average values of the Deep_PPI model via the independent test five times (from ‘Times = 1’ to ‘Times = 5’), as the ‘Overall’ row in Table 6. ‘5‐fold cross validation^#^’ means the average values of ‘Fold = 1’ to ‘fold = 5’ in the five‐fold cross validation, as the ‘Overall’ row in Table 7. The bold faced value denotes the high predictions in Table 9.

By observing the comparison results in Table 9, it can be seen that Deep_PPI outperformed DNN‐CTAC on the *C. elegans* and *E. coli* datasets by only regarding to the *AUC* value. More specifically, the *AUC* values of DNN_CTAC were 0.9790 ± 0.0018 and 0.9726 ± 0.0033, however, Deep_PPI (the independent test‐5times) could improve the corresponding value to 0.9923 ± 0.0063 and 0.9916 ± 0.0005, and Deep_PPI (5‐fold cross validation) also could improve the *AUC* value to 0.9923 ± 0.0063 and 0.9927 ± 0.0042. While on the *H. sapiens* dataset, DNN_CTAC (with *AUC* of 0.9875 ± 0.0010) performed slightly better than Deep_PPI (5‐fold cross validation, with *AUC* of 0.9859 ± 0.0034), but slightly worse than Deep_PPI (the independent test‐5times, with *AUC* of 0.9921 ± 0.0008). The underlying reason had been discussed in Section 4.3.3.

## 5 Conclusion

A broad spectrum of infectious diseases, cancer and neurodegenerative diseases are intricately linked to abnormal PPIs [41]. The correct identification of PPIs using DL methods aids in‐depth knowledge to understand their underlying complex biological mechanisms and new drug designing. In the present study, we developed a novel DL‐based PPI identifier named predictor called Deep_PPI. The proposed method outperformed existing advanced PPI predictors for all species in term of overall accuracy on training and testing datasets. We anticipate that, the proposed DL‐based PPI predicator not only helps pave the way towards discovering new PPI samples but also contributing potentially to the other bioinformatics fields.

Last but not least, we will further extend our research in three folds: (A) construction of publicly accessible webserver for the annotation of large‐scale PPI prediction (B) secondly, to encode the PPI sequences by using evolutionary‐based such as segmented position specific scoring matrix molecular string or multi‐view features [42] (C) third, we will design novel interpretable DL [43] or ensemble DL [44], and graph‐based transfer learning model, for targeting accurate PPI from sequences as well as from structural finger print data.

## Author Contributions

**Irfan Khan:** data collection, conceptualisation, methodology, software, experiments validation, formal analysis, investigation, writing–original draft. **Muhammad Arif:** formal analysis, validation, investigation, resources, visualisation, writing–review & editing, supervision. **Ali Ghulam:** formal analysis, validation, experiments, investigation, writing–review & editing, project management. **Somayah Albaradei:** data collection, formal analysis, validation, investigation, resources, visualisation. **Maha A. Thafar:** formal analysis, conceptualisation, methodology, writing–review & editing draft, funding, **Apilak Worachartcheewan:** validation, investigation, writing–review & editing draft, funding.

## Conflicts of Interest

The authors declare no conflicts of interest.

## Supporting information

Supplementary Material

## Data Availability

Data used in this study is publicly available.
