# Supplementary material for: Improved in Silico Identification of Protein‐Protein Interactions Using Deep Learning Approach
Source: IET Syst Biol. 2025 Apr 24;19(1):e70008. doi: 10.1049/syb2.70008 (PMC12021994; doi:10.1049/syb2.70008)
Supplement: Supplementary file 1 — Supplementary Material [file SYB2-19-e70008-s001.doc]

**Supplementary Material**

**Improved in Silico Identification of Protein-Protein Interactions using Deep Learning Approach**

Irfan Khan^1^, Muhammad Arif^2*^, Ali Ghulam^3^, Somayah Albaradei^4^, Maha A. Thafar^5^, Apilak Worachartcheewan^6*^

^1^Department of Computer Science, Abdul Wali Khan University Mardan, KPK, Pakistan

^2^College of Science and Engineering, Hamad Bin Khalifa University, Doha 34110, Qatar

^3^Information Technology Centre, Sindh Agriculture University, Tandojam, Sindh, 70060, Pakistan

^4^Department of Computer Science, Faculty of Computing and Information Technology, King Abdulaziz University, 21589, Jeddah, Saudi Arabia

^5^Computer Science Department, College of Computers and Information Technology, Taif University, Taif, Saudi Arabia

^6^Department of Community Medical Technology, Faculty of Medical Technology, Mahidol University, Bangkok, 10700, Thailand;

Corresponding author: * Email: [mfarif@hbku.edu.qa](mailto:mfarif@hbku.edu.qa); [apilak.woa@mahidol.edu](mailto:apilak.woa@mahidol.edu)

**Supplementary Text S1**

**The detailed explanations of four plots in Figure S1.**

There are some random parameters in the convolution layers of PPI_CNN. In order to evaluate the robustness of the PPI_CNN model, we trained the PPI_CNN model five times each training data of four datasets, and implemented test experiments on each independent test data part of four datasets. Herein, in **Figure S1**, we depicted the ROC and Recall-Precision curve of PPI_CNN via five times on the independent test data of four datasets, **Figure S1(A)** C.elegans dataset, **S1(B)** E.coli dataset, **S1(C)** H.sapiens dataset, **S1(D)** Human dataset. From four sub-plots, it is easily found that the PPI_CNN model could achieve quite high AUC and AP values. For instance, on the Human dataset, the AUCs of PPI_CNN are 0.9965, 0.9951, 0.9959, 0.9944, and 0.9946, and the APs of PPI_CNN are 0.9947, 0.9935, 0.9944, 0.9947, and 0.9923.

**Supplementary Text S2**

**The detailed explanations of four plots in Figure S2.**

In five-fold cross validation, each dataset is divided into five equal parts, train the PPI_CNN model on 4/5 parts of the data each time, and test the PPI_CNN model on the left 1/5 part of the data. After five cycles, each part of data was used as the training set for four times and as the test set for one time. We documented the evaluation of prediction results each time (i.e., “Fold=1”, “Fold=2”, “Fold=3”, “Fold=4”, and “Fold=5”). Herein, in **Figure S2**, we depicted the ROC and Recall-Precision curve of PPI_CNN via five-fold cross validation on four datasets, **Figure S2(A)** C.elegans dataset, **S2(B)** E.coli dataset, **S2(C)** H.sapiens dataset, **S2(D)** Human dataset. From four sub-plots, it is easily seen that the PPI_CNN model could achieve quite high AUC and AP values. For instance, on the Human dataset, the AUCs of PPI_CNN are 0.9957, 0.9940, 0.9961, 0.9927, and 0.9946, and the APs of PPI_CNN are 0.9945, 0.9910, 0.9957, 0.9945, and 0.9930.

**Supplementary Text S3**

**The detailed explanations of experiments results on four datasets listed in Table S1.**

**Table S1** listed the confusion matrix and three types of errors of PPI_CNN on the independent test data (via five times) of four datasets. The “Overall” row was the average and standard deviation values of corresponding evaluation indices from “Times=1” to “Times=5”. By observing the results on four datasets, we could see that the PPI_CNN model’s prediction performance was quite stable. Taking the Human dataset as an example, the *TP* and *TN* values of PPI_CNN are 3632(49.68%) and 3565(48.76%) (Times=1); 3614(49.43%) and 3556(48.64%) (Times=2); 3620(49.51%) and 3561(48.71%) (Times=3); 3607(49.34%) and 3565(48.76%) (Times=4); 3621(49.53%) and 3549(48.54%) (Times=5), respectively. Accordingly, the “Overall” values of *TP* and *TN* are 3618.8±9.2574 and 3559.2±6.7971.

Except for the *TP*, *TN*, *FP*, and *FN* values, we also calculated the corresponding *ER*, *FPR*, and *FNR* values built on the confusion matrix values (The formula was given in the section 2.3 of the manuscript). As seen, the PPI_CNN model could achieve quilt low errors (i.e., *ER*, *FPR*, and *FNR*). For instance, on the C.elegans dataset, the “Overall” values of *ER*, *FPR*, and *FNR* are 0.0042±0.0021, 0.0089±0.0044, and 0.0156±0.0095.

**Supplementary Text S4**

**The detailed explanations of experiments results on four datasets listed in Table S2.**

**Table S2** listed the Confusion matrix and three types of errors of PPI_CNN via 5-fold cross validation on four datasets. The “Overall” row was the average and standard deviation values of corresponding evaluation indices from “Fold=1” to “Fold=5”. By observing the results on four datasets, we could see that the PPI_CNN model’s prediction performance was quite stable. Taking the E.coli dataset as an example, the *TP* and *TN* values of PPI_CNN are 1352(48.60%) and 1380(49.60%) (Fold=1); 1338(48.09%) and 1390(49.96%) (Fold=2); 1332(47.88%) and 1390(49.96%) (Fold=3); 1340(48.17%) and 1378(49.53%) (Fold=4); 1318(47.38%) and 1376(49.46%) (Fold=5), respectively. Accordingly, the “Overall” values of *TP* and *TN* are 1336±12.4097 and 1382.8±6.7231.

Except for the *TP*, *TN*, *FP*, and *FN* values, we also calculated the corresponding *ER*, *FPR*, and *FNR* values built on the confusion matrix values (The formula was given in the section 2.3 of the manuscript). As seen, the PPI_CNN model could achieve quilt low errors (i.e., *ER*, *FPR*, and *FNR*). For instance, on the C.elegans dataset, the “Overall” values of *ER*, *FPR*, and *FNR* are 0.0037±0.0036, 0.0074±0.0072, and 0.0352±0.0101.

**Supplementary Figure S1**


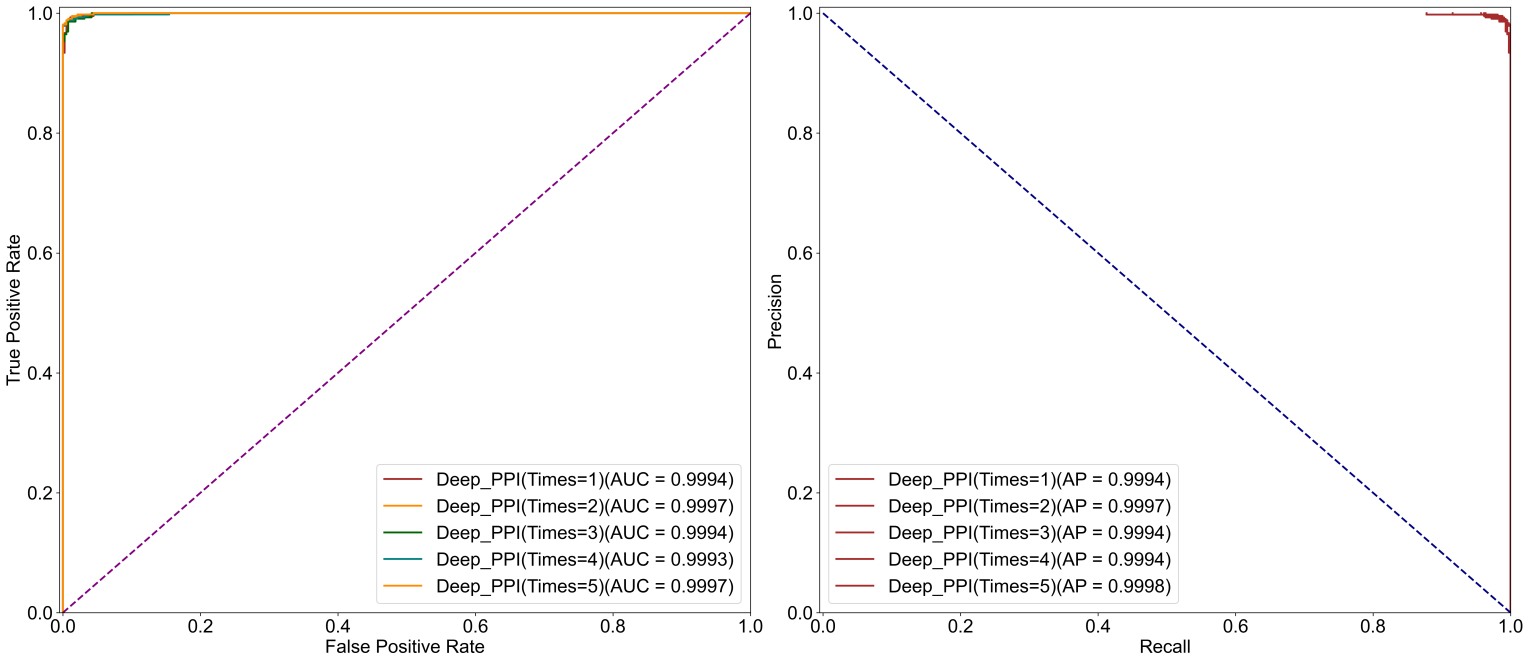


**(A)** C.elegans dataset

**
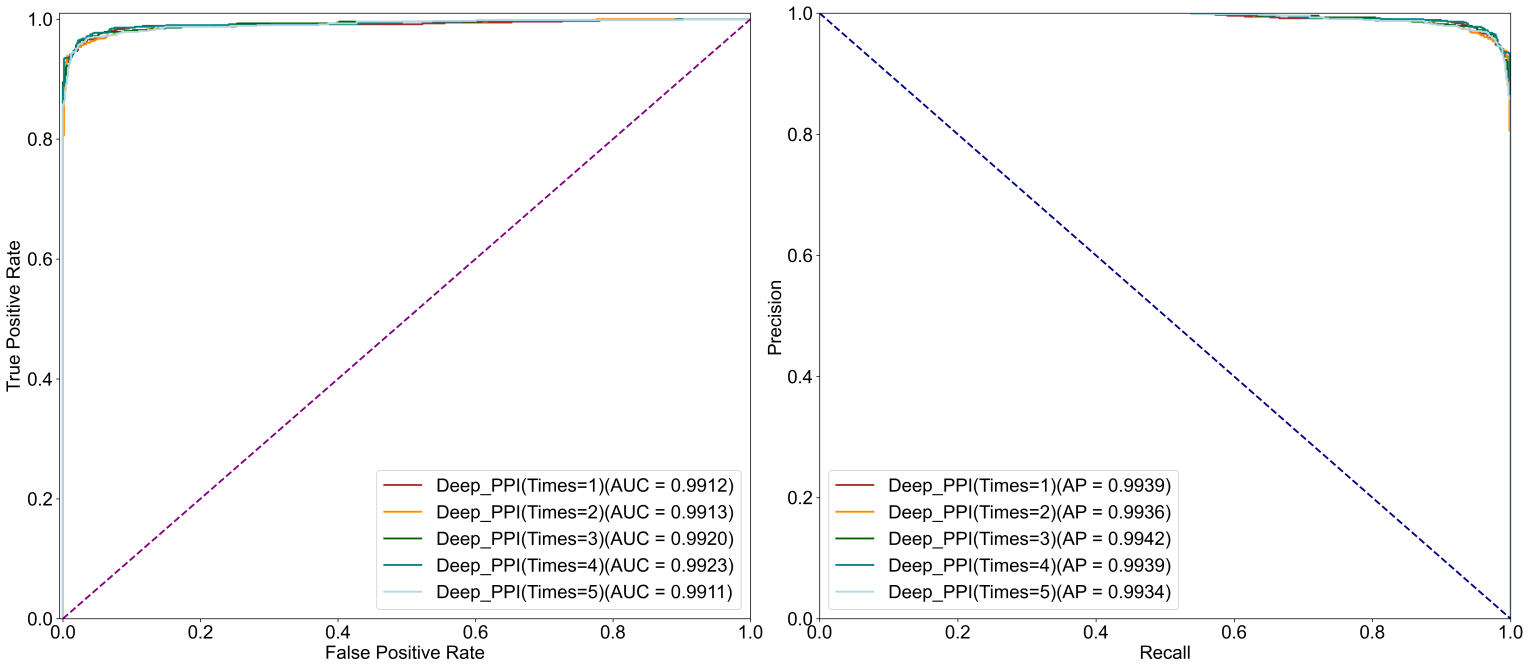
**

**(B)** E.coli dataset


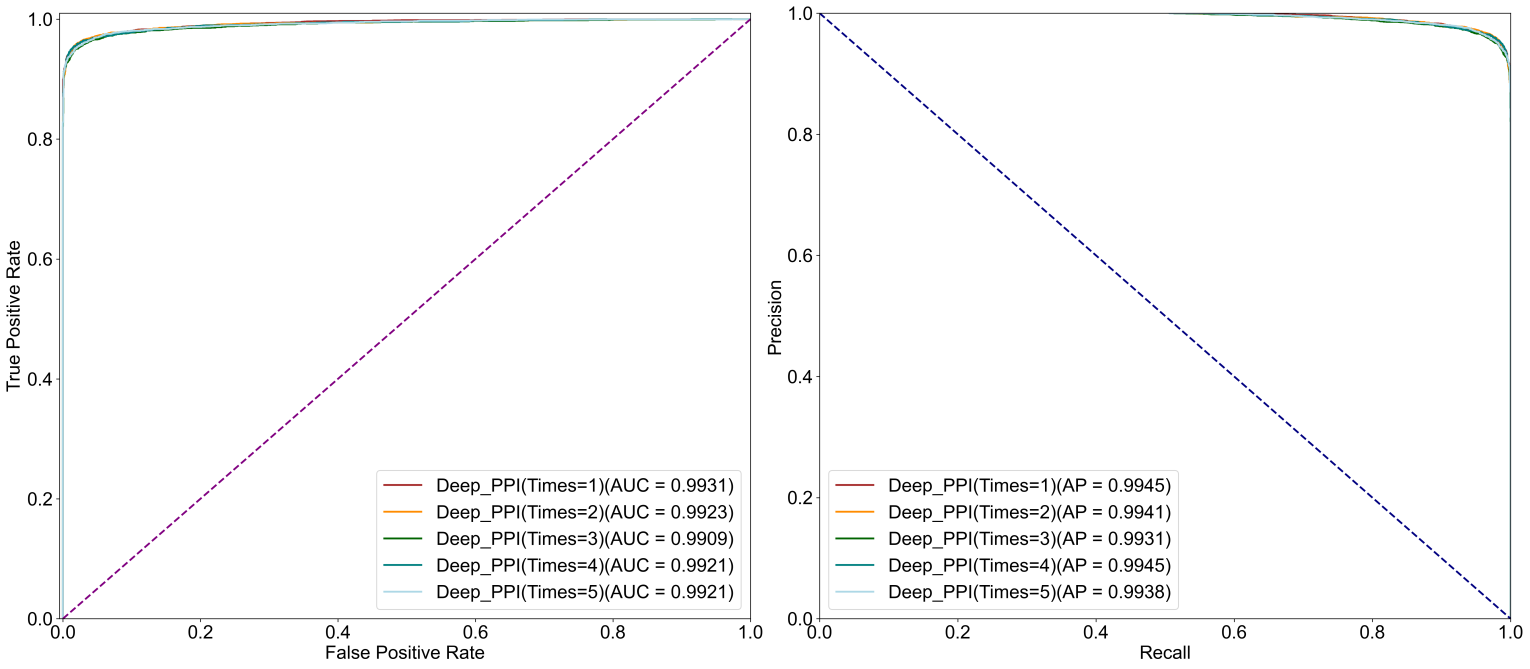


**(C)** H.sapiens dataset


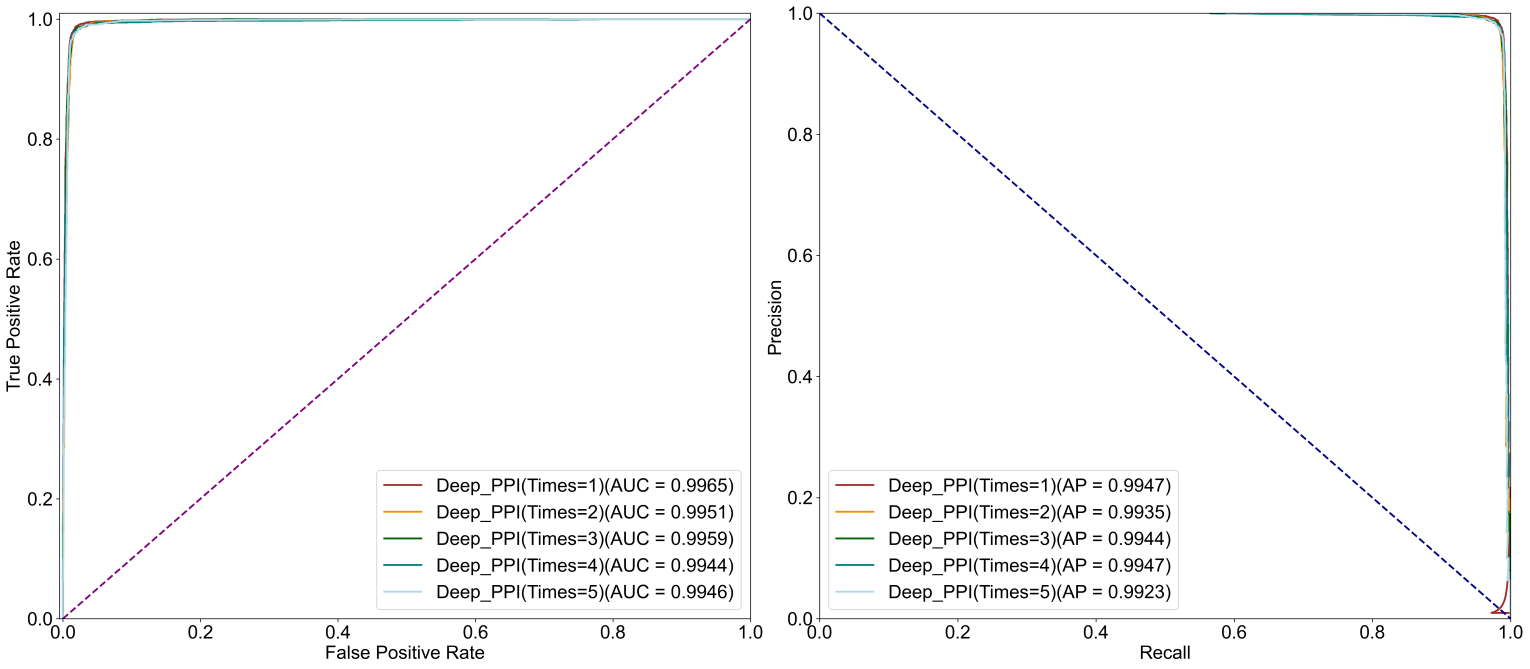


**(D)** Human dataset

**Figure S1**. ROC and Recall-Precision curve of PPI_CNN via five times on the independent test data of four datasets. Note: “Times=1、2、3、4 and 5” means testing the PPI_CNN model first、second、third、fourth and fifth times.

**Supplementary Figure S2**


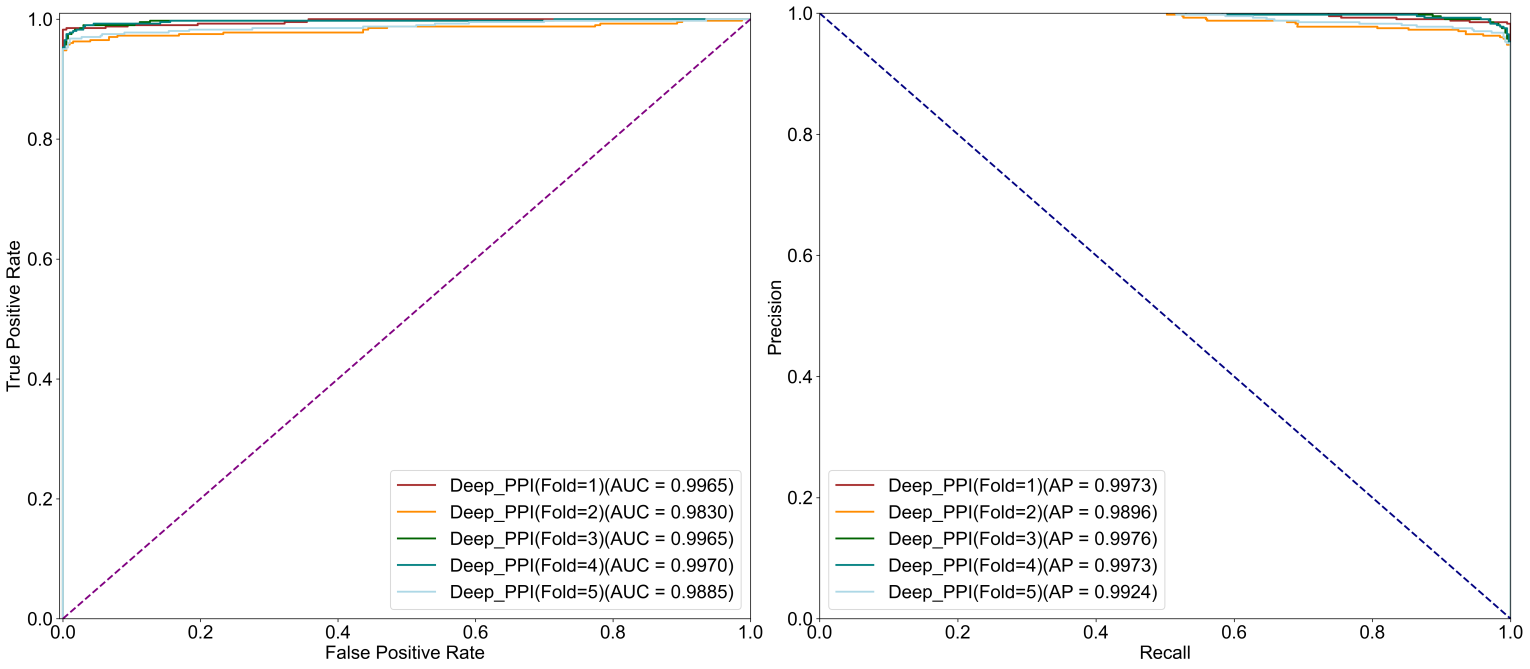


**(A)** C.elegans dataset


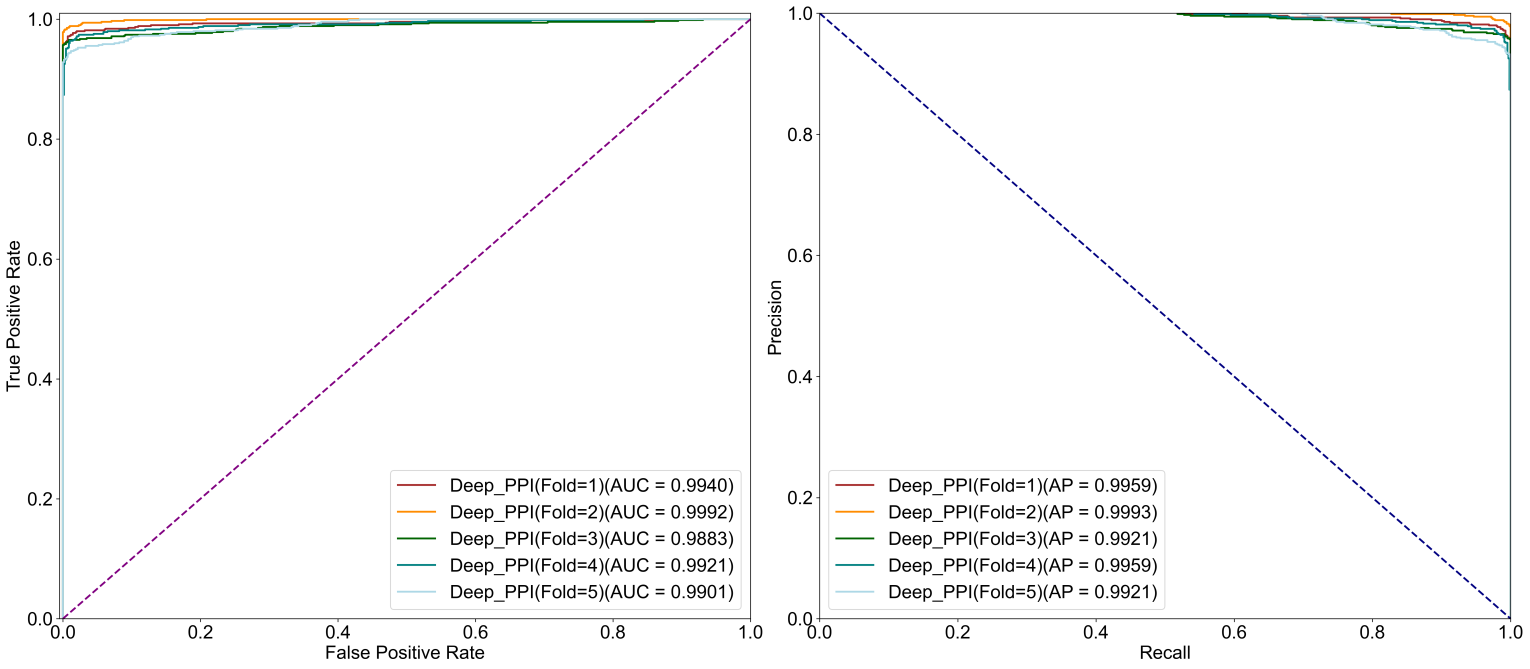


**(B)** E.coli dataset


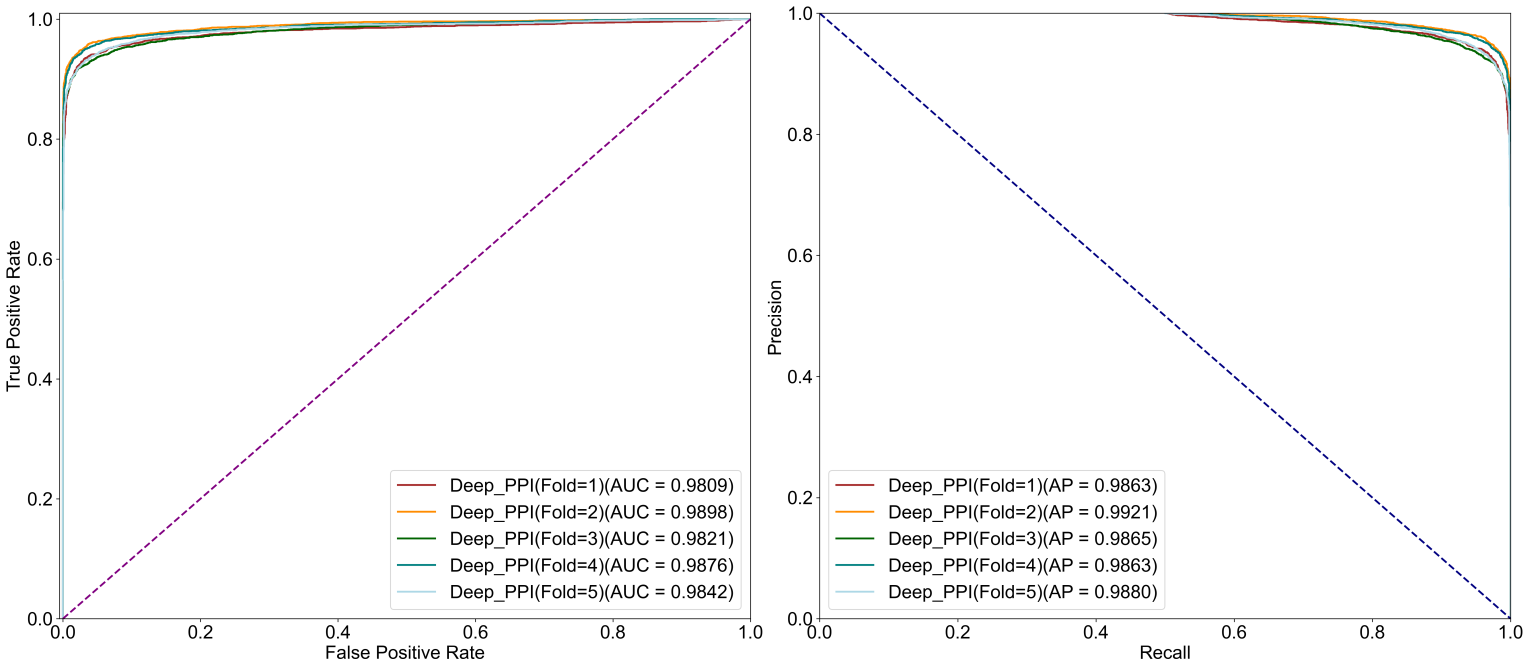


**(C)** H.sapiens dataset


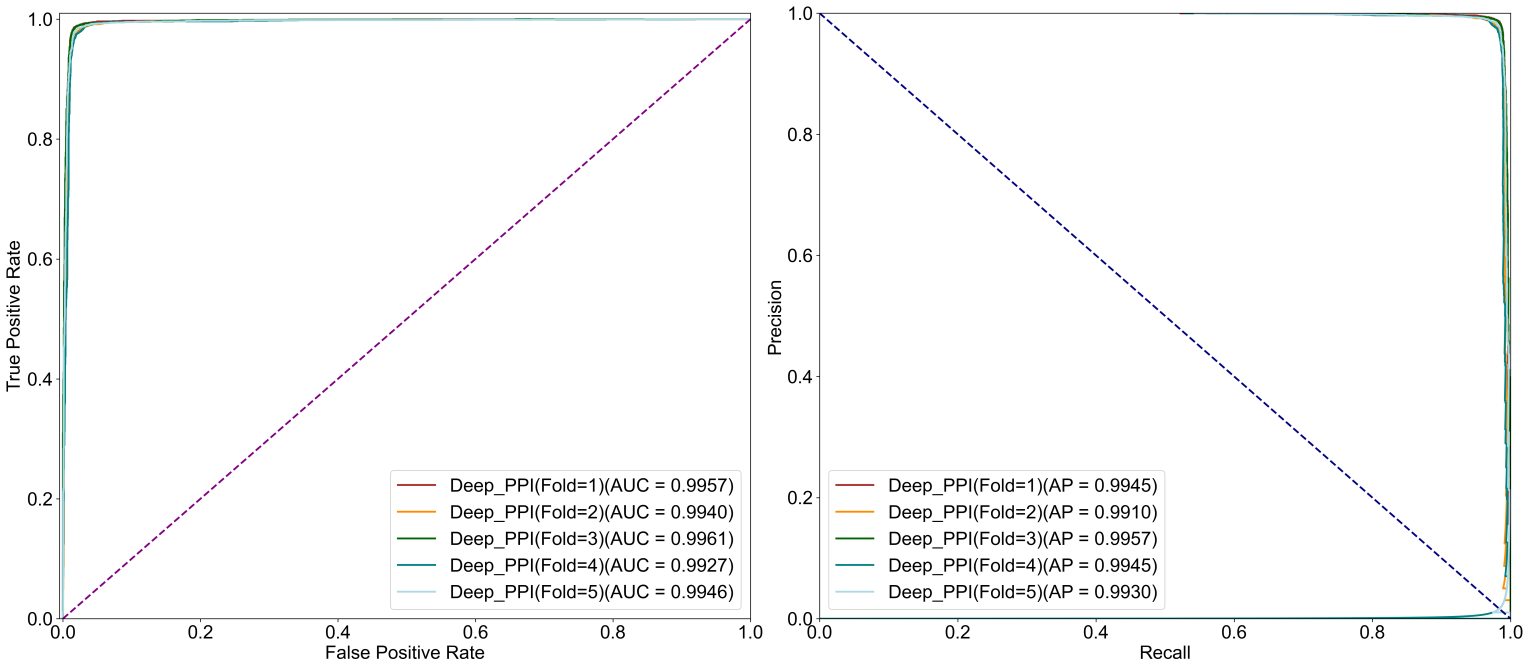


**(D)** Human dataset

**Figure S2**. ROC and Recall-Precision curve of PPI_CNN via 5-fold cross-validation on four datasets. Note: “Fold=1、2、3、4 and 5” means testing the PPI_CNN model first、second、third、fourth and fifth-fold cycle in the five-fold cross validation.

**Supplementary Table S1**

**Table S1.** Confusion matrix and three types of errors of PPI_CNN on the independent test data (via five times) of four datasets

| Dataset | Times | *TP* | *TN* | *FP* | *FN* | *ER* | *FPR* | *FNR* |
| --- | --- | --- | --- | --- | --- | --- | --- | --- |
| C.elegans | Times=1 | 421(52.23%) | 377(46.77%) | 5(0.62%) | 3(0.37%) | 0.0062 | 0.0131 | 0.0071 |
|  | Times=2 | 419(51.99%) | 379(47.02%) | 3(0.37%) | 5(0.62%) | 0.0037 | 0.0079 | 0.0118 |
|  | Times=3 | 411(50.99%) | 379(47.02%) | 3(0.37%) | 13(1.61%) | 0.0037 | 0.0079 | 0.0307 |
|  | Times=4 | 416(51.61%) | 381(47.27%) | 1(0.12%) | 8(0.99%) | 0.0012 | 0.0026 | 0.0189 |
|  | Times=5 | 420(52.11%) | 377(46.77%) | 5(0.62%) | 4(0.50%) | 0.0062 | 0.0131 | 0.0094 |
|  | Overall | 417.4±4.0373 | 378.6±1.6733 | 3.4±1.6733 | 6.6±4.0373 | 0.0042±0.0021 | 0.0089±0.0044 | 0.0156±0.0095 |
| E.coli | Times=1 | 683(49.10%) | 660(47.45%) | 19(1.37%) | 29(2.08%) | 0.0137 | 0.0280 | 0.0407 |
|  | Times=2 | 678(48.74%) | 666(47.88%) | 13(0.93%) | 34(2.44%) | 0.0093 | 0.0191 | 0.0478 |
|  | Times=3 | 689(49.53%) | 656(47.16%) | 23(1.65%) | 23(1.65%) | 0.0165 | 0.0339 | 0.0323 |
|  | Times=4 | 682(49.03%) | 659(47.38%) | 20(1.44%) | 30(2.16%) | 0.0144 | 0.0295 | 0.0421 |
|  | Times=5 | 681(48.96%) | 664(47.74%) | 15(1.08%) | 31(2.23%) | 0.0108 | 0.0221 | 0.0435 |
|  | Overall | 682.6±4.0373 | 661±4 | 18±4 | 29.4±4.0373 | 0.0129±0.0029 | 0.0265±0.0059 | 0.0413±0.0057 |
| H.sapiens | Times=1 | 3532(47.69% ) | 3631(49.03%) | 74(1.00%) | 169(2.28%) | 0.0100 | 0.0200 | 0.0457 |
|  | Times=2 | 3528(47.64%) | 3647(49.24%) | 58(0.78%) | 173(2.34%) | 0.0078 | 0.0157 | 0.0467 |
|  | Times=3 | 3529(47.65%) | 3615(48.81%) | 90(1.22%) | 172(2.32%) | 0.0122 | 0.0243 | 0.0465 |
|  | Times=4 | 3531(47.68%) | 3657(49.38%) | 48(0.65%) | 170(2.30%) | 0.0065 | 0.0130 | 0.0459 |
|  | Times=5 | 3507(47.35%) | 3645(49.22%) | 60(0.81%) | 194(2.62%) | 0.0081 | 0.0162 | 0.0524 |
|  | Overall | 3525.4±10.4067 | 3639±16.3095 | 66±16.3095 | 175.6±10.4067 | 0.0089±0.0022 | 0.0178±0.0044 | 0.0474±0.0028 |
| Human | Times=1 | 3632(49.68%) | 3565(48.76%) | 71(0.97%) | 43(0.59%) | 0.0097 | 0.0195 | 0.0117 |
|  | Times=2 | 3614(49.43%) | 3556(48.64%) | 80(1.09%) | 61(0.83%) | 0.0109 | 0.0220 | 0.0166 |
|  | Times=3 | 3620(49.51%) | 3561(48.71%) | 75(1.03%) | 55(0.75%) | 0.0103 | 0.0206 | 0.0150 |
|  | Times=4 | 3607(49.34%) | 3565(48.76%) | 71(0.97%) | 68(0.93%) | 0.0097 | 0.0195 | 0.0185 |
|  | Times=5 | 3621(49.53%) | 3549(48.54%) | 87(1.19%) | 54(0.74%) | 0.0119 | 0.0239 | 0.0147 |
|  | Overall | 3618.8±9.2574 | 3559.2±6.7971 | 76.8±6.7971 | 56.2±9.2574 | 0.0105±0.0009 | 0.0211±0.0019 | 0.0153±0.0025 |

**Supplementary Table S2**

**Table S2**. Confusion matrix and three types of errors of PPI_CNN via 5-fold cross validation on four datasets

| Dataset | Fold | *TP* | *TN* | *FP* | *FN* | *ER* | *FPR* | *FNR* |
| --- | --- | --- | --- | --- | --- | --- | --- | --- |
| C.elegans | Fold=1 | 776(48.14%) | 806(50.00%) | 0(0.00%) | 30(1.86%) | 0.0000 | 0.0000 | 0.0372 |
|  | Fold=2 | 768(47.64%) | 802(49.75%) | 4(0.25%) | 38(2.36%) | 0.0025 | 0.0050 | 0.0471 |
|  | Fold=3 | 790(49.01%) | 792(49.13%) | 14(0.87%) | 16(0.99%) | 0.0087 | 0.0174 | 0.0199 |
|  | Fold=4 | 774(48.01%) | 804(49.88%) | 2(0.12%) | 32(1.99%) | 0.0012 | 0.0025 | 0.0397 |
|  | Fold=5 | 780(48.39%) | 796(49.38%) | 10(0.62%) | 26(1.61%) | 0.0062 | 0.0124 | 0.0323 |
|  | Overall | 777.6±8.1731 | 800±5.831 | 6±5.831 | 28.4±8.1731 | 0.0037±0.0036 | 0.0074±0.0072 | 0.0352±0.0101 |
| E.coli | Fold=1 | 1352(48.60%) | 1380(49.60%) | 10(0.36%) | 40(1.44%) | 0.0036 | 0.0072 | 0.0287 |
|  | Fold=2 | 1338(48.09%) | 1390(49.96%) | 0(0.00%) | 54(1.94%) | 0.0000 | 0.0000 | 0.0388 |
|  | Fold=3 | 1332(47.88%) | 1390(49.96%) | 2(0.07%) | 58(2.08%`) | 0.0007 | 0.0014 | 0.0417 |
|  | Fold=4 | 1340(48.17%) | 1378(49.53%) | 14(0.50%) | 50(1.80%) | 0.0050 | 0.0101 | 0.0360 |
|  | Fold=5 | 1318(47.38%) | 1376(49.46%) | 16(0.58%) | 72(2.59%) | 0.0058 | 0.0115 | 0.0518 |
|  | Overall | 1336±12.4097 | 1382.8±6.7231 | 8.4±7.1274 | 54.8±11.7132 | 0.003±0.0026 | 0.006±0.0051 | 0.0394±0.0084 |
| H.sapiens | Fold=1 | 6914(46.68%) | 7178(48.46%) | 228(1.54%) | 492(3.32%) | 0.0154 | 0.0308 | 0.0664 |
|  | Fold=2 | 6954(46.95%) | 7276(49.12%) | 130(0.88%) | 452(3.05%) | 0.0088 | 0.0176 | 0.0610 |
|  | Fold=3 | 7022(47.41%) | 6852(46.26%) | 554(3.74%) | 384(2.59%) | 0.0374 | 0.0748 | 0.0518 |
|  | Fold=4 | 6938(46.84%) | 7266(49.05%) | 140(0.95%) | 468(3.16%) | 0.0095 | 0.0189 | 0.0632 |
|  | Fold=5 | 6898(46.58%) | 7152(48.29%) | 252(1.70%) | 508(3.43%) | 0.0170 | 0.0340 | 0.0686 |
|  | Overall | 6953±51.6785 | 7136.5±197.8307 | 269±197.8855 | 453±51.6785 | 0.0182±0.0134 | 0.0363±0.0267 | 0.0612±0.007 |
| Human | Fold=1 | 7198(49.23%) | 7190(49.17%) | 106(0.72%) | 128(0.88%) | 0.0072 | 0.0145 | 0.0175 |
|  | Fold=2 | 7170(49.04%) | 7144(48.86%) | 152(1.04%) | 156(1.07%) | 0.0104 | 0.0208 | 0.0213 |
|  | Fold=3 | 7224(49.41%) | 7166(49.01%) | 130(0.89%) | 102(0.70%) | 0.0089 | 0.0178 | 0.0139 |
|  | Fold=4 | 7168(49.02%) | 7118(48.68%) | 178(1.22%) | 158(1.08%) | 0.0122 | 0.0244 | 0.0216 |
|  | Fold=5 | 7226(49.42%) | 7110(48.63%) | 186(1.27%) | 100(0.68%) | 0.0127 | 0.0255 | 0.0137 |
|  | Overall | 7197±32.3522 | 7134.5±25.5278 | 161.5±25.5278 | 129±32.3522 | 0.011±0.0017 | 0.0221±0.0035 | 0.0176±0.0044 |

**Supplementary Table S3. Hyperparameters search range different machine learning classifiers.**

| **Method** | **Parameter** | **Tested values** |
| --- | --- | --- |
| RF | Number of estimators (n_estimators^[a]^) | 60–1000 with an interval of 20 |
|  | Maximum number of features considered per split (max_features^[a]^) | 1–20 with an interval of 1, |
|  |  |  |
| DT | Minimum number of samples required to split an internal node (min_samples_split^[a]^) | 2–10 with an interval of 1 |

Column 1 represents the ML algorithms. Column 2 and 3 represent the parameter name and the tested values, respectively. ^[a]^ Parameter name in the Scikit-learn implementation.
